# Knowledge, attitude, and practice regarding child maltreatment amongst Iranian medical students through internship course: an 18-month longitudinal study

**DOI:** 10.1186/s12875-023-01988-9

**Published:** 2023-01-31

**Authors:** Mahsa Boroon, Saba Mokhtari, Marzieh Nojomi, Fatemeh Hadi, Shiva Soraya, Mohammadreza Shalbafan

**Affiliations:** 1 Mental Health Research Center, Psychosocial Health Research Institute, Department of Psychiatry, School of Medicine, Iran University of Medical Sciences, Tehran, Iran; 2 Department of Psychiatry, University of Social Welfare and Rehabilitation Sciences, Tehran, Iran; 3 Preventive Medicine and Public Health Research Center, Psychosocial Health Research Institute, Department of Community and Family Medicine, Iran University of Medical Sciences, Tehran, Iran; 4 School of Medicine, Shahid Beheshti University of Medical Sciences, Tehran, Iran; 5 Research Center for Addiction and Risky Behaviors, Psychosocial Health Research Institute, Department of Psychiatry, School of Medicine, Iran University of Medical Sciences, Tehran, Iran

**Keywords:** Child maltreatment, Medical students, Medical education, Iran

## Abstract

**Background:**

Child maltreatment has been a major worldwide problem and has remained a persistent public health challenge in all countries. Physicians have a significant role in the prevention and intervention of child maltreatment. An educated physician that can effectively identify and report child maltreatment can fill one of the most significant gaps in reducing child abuse.

This study was performed to examine the knowledge, attitude, and practice regarding child maltreatment among Iranian medical students through an internship course.

**Method:**

All the medical students (235 students) of the Iran University of Medical Sciences who passed the internship entrance exam in the 2019–2020 academic year participated in this study. The participants completed a 49-item scale questionnaire built by combining three other validated questionnaires that evaluate their attitude, knowledge, and practice skills in the first month of their internship course and then completed the same questionnaire 18 months later, at the end-point of the internship period.

**Results:**

One-hundred thirty nine (59.1%) participants were female, and 96 (40.9%) were male. The mean age of the subjects was 24.35 ± 0.76. Only 7 (3%) of them formerly received any education about child maltreatment. There was a significant improvement in scores of the knowledge of prevention (*p*-value = 0.001), the practice of prevention (*p*-value < 0.001) and the general subscale of the practice section (*p*-value < 0.001) during the internship course. However, the performance of participants decreased significantly in the subscales of the attitude towards diagnosis (*p*-value = 0.001) and the attitude towards reporting (*p*-value < 0.001) of child maltreatment. At the end of the study, the result of graduated physicians was: The total knowledge and attitude of participants were satisfactory, and the majority were at reasonable levels. Although in the practice subscale, 70.6% of the participants didn’t identify, 84.7% didn’t refer, and 86.4% didn’t report a child abuse case in the past year.

**Conclusion:**

The knowledge and attitude of Iranian physicians regarding child maltreatment are at a satisfactory level. Although, the practice level has deficiencies. In addition, our findings show that Iranian interns have a shallow experience regarding child maltreatment, particularly despite the higher scores in attitude and knowledge, which can be the reason for deficiencies in the practice level.

**Supplementary Information:**

The online version contains supplementary material available at 10.1186/s12875-023-01988-9.

## Background

Child maltreatment or abuse has been a major worldwide problem and has remained a persistent public health challenge in all countries, regardless of their income level [1]. The World Health Organization (WHO) defines child abuse as any interaction in the context of a relationship of responsibility, trust, or power that violates children’s rights and results in harm to children’s dignity, development, or life [2]. Iran Law on Protection of Children and Adolescents (passed in 2002) defines child maltreatment as “abuse of any kind to children and adolescents which causes physical, mental, or moral harms or endangers their physical or mental health” [3]. Child maltreatment includes neglect, and physical, sexual and emotional abuse [4].

A long record of research has studied the negative outcome of child maltreatment. Alongside the short-term consequences [5–8], child abuse can cause different long-term impacts, such as depression, anxiety and eating disorders, antisocial behaviors, somatic symptom disorders, suicidal and self-harm behaviors, and substance use disorder [9–14].

WHO reports that internationally, 3 of 4 children aged 2–4 years suffer from child maltreatment, and 1 in 5 women and 1 in 13 men have been sexually abused as a child. Additionally, many studies have shown that child abuse rates have risen during the COVID-19 pandemic [15]. However, these numbers underestimate the actual size of the issue due to a lack of reports.

Iran has a population of more than 80 million with a high rate of the youth population. Around 30% of Iran’s population is under 18 years old [16, 17]. In reports of a systematic review and meta-analysis performed in 2014 on child abuse in Iran, the pooled estimate of the prevalence of child emotional abuse was 64.5%, 43.59% for physical abuse, and 40.94% for neglect [18]. Reporting child maltreatment is not mandated in Iran, and there is no protocol for it. Hence, many cases are missed. In addition, due to religious beliefs and social and cultural context, reporting child abuse is challenging in Iran [19].

Physicians have a significant role in preventing and intervening of child maltreatment [20]. They have regular contact with children during well-child visits and vaccination appointments, which is the most minor health-care action that all the parents, regardless of their persistence, should perform [20]. An educated physician that can effectively identify and report child maltreatment can fill one of the most significant gaps in reducing child abuse [21]. Some studies have shown several factors that cause low rates of reporting child maltreatment among medical professionals and medical students. These factors and characteristics consist of a lack of specified training, feeling unprepared and under-trained, fear of having a negative impact, and being unsure of the adequacy of facts [22–25].

Medical training in Iran takes seven years and consists of the following: 1) Two years of Basic Science Training, 2) Three and half years of clinical theoretical and observational training followed by a "Pre-Internship Examination,” 3) Eighteen months of Internship. In the internship course, medical students work as interns in specialized hospital wards with the supervision of board-certified specialists who provide expertise, professional and ethical practice, and role modeling in delivering patient care [26, 27]. The internship period of pediatrics lasts three months and as explained in the educational curriculum of general medicine interns learn the skills of taking history, medical examination, diagnosis and differential diagnosis, treatment (evidence-based and based on guidelines), and common procedures of common and important pediatric problems during this period. Iranian interns work in educational hospitals and are the first members of the healthcare system who visit the patients and are involved in all processes of diagnosis and treatment. 50% of this education is in pediatric wards, and the other 50% in pediatric clinics, emergency rooms, and pediatric consultations [28]. Additional to the pediatric curriculum, Iranian interns pass 40 h of education on domestic violence, and 20 h of this program are regarding child maltreatment [29].

Clinical internships includes courses that enable learning in the natural environment and turning theoretical knowledge into practical skills [30]. Internship courses for medical students in teaching hospitals have a crucial role in training particular subjects like prevention strategies, social accountability, and managing skill [31, 32].

Several studies have been conducted on medical professionals’ attitudes, knowledge, and experience of regarding child maltreatment in different countries. All of these studies have shown unsatisfactory results in at least one of the attitude, knowledge, management, or practice areas [19, 33–39]. The study of Sahebihagh et al. [39] evaluated the knowledge, attitude, and practice regarding child abuse among community health workers in Tabriz and reported good knowledge, favorable attitude, and poor performance regarding child maltreatment. Jahanimoghadam et al. [38] performed a study on knowledge, attitude, and practice regarding child abuse among Iranian dentists and pedodontists. They reported moderate knowledge, poor attitude, and moderate practice regarding child abuse. However, there is no study on this matter among Iranian physicians, particularly interns.

This study was performed to examine the change in the knowledge, attitude, and practice regarding child maltreatment among Iranian medical students through internship course, and the outcome of knowledge, attitude, and practice in regards to child maltreatment amongst recently graduated physicians in Iran University of Medical Sciences, Tehran, Iran, by a longitudinal study.

## Materials and methods

### Design and participants

The subjects of this longitudinal study consisted of medical students of the Iran University of Medical Sciences. To determine the 0.2 pints difference in knowledge score before and after the internship course, with type one error of 0.05 and power of 80%, at least sample size was calculated 199. For convenience, we selected all medical students who passed the internship entrance exam in the 2019–2020 academic year with a size of 235.

The exclusion criteria were: 1) refusal to participate, 2) history of passing the internship course in another country, 3) history of having another clinical academic education.

The participants completed the questionnaire about their attitude, knowledge, and practice skills in the first month of their internship course. Then they completed the same questionnaire 18 months later, at the end-point of the internship period.

### Assessment tool

The questionnaire on attitude, knowledge, and practice skills toward child maltreatment was used to gather the data.

In the study of Deshpande et al., a questionnaire with 18 items was developed and used. The reliability and validity of this questionnaire were examined and reported in the same study [40]. In the study of Kara et al., a 25 items scale was developed. The Cronbach alpha values were reported to be 0.68, 0.72, and 0.77 for “Symptoms,” “History,” and “Examination,” respectively [41]. In the study of Lane et al. a 38-item questionnaire was developed and used to evaluate the knowledge, comfort, and competence of pediatricians [42]. In this study, we used the questionnaire built into the study of Abouei et al. upon the three questionnaires mentioned above.

In the study of Abouei et al. with the title ‘Knowledge, attitude, and practice in regards to child maltreatment among Emergency Medicine residents in Tehran’**,** these three questionnaires were combined, and after deleting the duplicate questions, a 49-item scale to evaluate the knowledge, attitude and practice of medical professionals has been developed. They reported acceptable face validity and good internal consistency for this newly developed questionnaire (Cronbach’s alpha = 0.71) [43].

The subscales of this questionnaire are 1) Knowledge subscale: 20 self-report “yes/no” questions with 46 scores: Eight questions about knowledge of prevention (16 scores), eight questions about knowledge of diagnosis (24 scores), two questions about knowledge of treatment (two scores) and two questions about knowledge of reporting (four scores). 2) Attitude subscale: 15 self-report Likert scale with 33 scores: Four questions about the attitude towards prevention (eight scores), five questions about the attitude towards diagnosis (15 scores), two questions about the attitude towards treatment (two scores), and four questions about the attitude towards reporting (eight scores). 3) Practice subscale: 12 self-report questions (nine Likert scales and 3 “yes/no” questions): one question about the practice of prevention (two scores), two questions about the practice of diagnosis (five scores), two questions about the practice of treatment (two scores), four questions about the practice of reporting (six scores) and three “yes/no” questions generally about the practice with four scores.

The knowledge subscale of the questionnaire was categorized into three levels: 1) poor: a score of less than 50%, 2) moderate: a score of 50‒70%, 3) good: a score over 70% [38, 44, 45].

### Statistical analysis

For statistical analyses, we used SPSS 24. To check the normality of changes in each subscale’s scores, we used the Kolmogorov–Smirnov test. For all variables, the *P*-value was less than 0.05. Therefore, we used the Wilcoxon matched-pair signed rank-rank test to assess the changing in each subscale’s questionnaire scores during the study period. For evaluation of the difference in subscale scores, we used the Mann Whitney U test across gender and marital status. For testing the significant differences, we considered *P*-values less than 0.05.

## Results

### Participants

A total of 235 medical students participated in the study. 139 (59.1%) of the participants were female, and 96 (40.9%) were male. The mean age of the subject was 24.35 ± 0.76. 212 (90.2%) of participants were single, and only seven (3%) of them formerly received any education about child maltreatment (at the start of the study).

### Knowledge, attitude, and practice of recently graduated medical practitioners toward child maltreatment

The frequency distribution of participants’ answers to each item of knowledge, attitude, and practice regarding the child maltreatment scale, at the end of the internship course is presented in Appendix 1.

We evaluated the answers of our participants to each item of knowledge, attitude, and practice regarding child maltreatment, at the end of the internship course (at the end of medical school).

More than 77% percent (*N* = 183) of participants correctly answered seven of eight questions on the knowledge of prevention subscale. Near all of the participants)98.3%, (*N* = 231) ( correctly reported that children who have been abused generally delay telling someone about the incident, 81.7% (*N* = 191) identified that in most cases, the abuser is someone the child knows well, and 68.5% agreed that child abuse can occur in high socioeconomic groups and is not primarily associated with poverty. Regarding signs of child maltreatment, 91.1% (*N* = 214) of the participants correctly identified wetting the bed after gaining toilet education, 88.1% (*N* = 207) detected fear of going home or of the parents, 96.6% (*N* = 227) identified vague history, and 56.6% (*N* = 133) reported burn signs. 59.1% (*N* = 138) of the participants didn’t know that bruises over bony prominences are not a sign of abuse. More than 90% of the participants answered both questions of knowledge of reporting questions correctly.

The frequency distribution of participants’ knowledge levels (poor, moderate, and good) towards child maltreatment at the end of the internship course (end of medical training) is shown in Table 1. According to this table, the total knowledge of 209 (88.93%) participants is good, and none of the subjects are at a poor level. Also, there are no participants with a poor level of knowledge of prevention and diagnosis. In the subscale of knowledge of diagnosis, 196 (83.41%) participants are at a good level. Only nine (3.82%) participants are at a poor level in the subscale of the knowledge of treatment, and four (1.7%) participants in the subscale of the knowledge of reporting are at a poor level. In the subscale of the knowledge of treatment, 77 (32.76%) are at a moderate level, and 149 (63.4%) participants are at a good level. In the subscale of the knowledge of reporting, 209 (88.93%) participants are at a good level.

**Table 1:** Frequency distribution of participants’ knowledge level regarding child maltreatment, at the end of internship course

			**Frequency**	**Percent**
**Knowledge**	**Prevention**	**Poor**	0	0
		**Moderate**	27	11.4
		**Good**	208	88.5
	**Diagnosis**	**Poor**	0	0
		**Moderate**	39	16.5
		**Good**	196	83.4
	**Treatment**	**Poor**	9	3.8
		**Moderate**	77	32.7
		**Good**	149	63.4
	**Reporting**	**Poor**	4	1.7
		**Moderate**	22	9.3
		**Good**	209	88.9
	**Total**	**Poor**	0	0
		**Moderate**	26	11
		**Good**	209	88.9

In the subscale of Attitude towards prevention of child abuse, nearly all participants (97.4%, *N* = 228) strongly agreed/ agreed with the importance of child abuse education, and (94%, *N* = 220) revised the child abuse laws. 86.4% (*N* = 203) of the participants strongly agreed/ agreed to attend workshops or symposiums to improve their knowledge regarding child abuse. Although 67.7% (*N* = 159) strongly agreed/ agreed that due to children's fear and embarrassment of reporting child abuse, even allowing the child to contact child abuse prevention centers are ineffective in reducing its rate.

In the attitude towards the diagnosis of child abuse statements, 94.9% (*N* = 223) of participants strongly agreed/ agreed that a physician should evaluate a child suspected of child abuse at the first visit. 52.8% (*N* = 124) of the participants strongly agreed/ agreed to refer a case suspected of child abuse to a pediatrician, and 86.6% (*N* = 203) to a psychiatrist.

More than 90% of participants strongly agreed/ agreed that family education and psychological and social support for families are ways of treatment of child abuse.

In the subscale of attitude towards reporting, the most reasons that the participants strongly agree/ agree for not reporting suspicious cases of child abuse were: 46.8% (*N* = 109) possible effect on the child, 41.3% (*N* = 97) fear of anger from parents and family, 37.1% (*N* = 87) possible effect on practice and 29% (*N* = 68) no legal obligation or authority to report.

In the practice subscale, 70.2% (*N* = 164) of the participants had never attended a child-abuse conference in the last three years. 70.6% (*N* = 165) of the participants didn’t identify, 84.7% (*N* = 199) didn’t refer, and 86.4% (*N* = 203) didn’t report a case of child abuse in the past year. 55.3% (*N* = 129) of the physicians had been trained in child abuse before graduation, but 85.1% (*N* = 199) believed the methods they taught to diagnose child abuse are insufficient.

### Change of knowledge, attitude, and practice of medical students toward child maltreatment during internship course

There is a significant increase in the mean scores of the knowledge of prevention (*p*-value = 0.001), the practice of prevention (*p*-value < 0.001) and the general subscale of the practice section (*p*-value < 0.001) during the internship course. However, the performance of participants decreased significantly in the mean scores of the subscales of the attitude towards diagnosis (*p*-value = 0.001) and the attitude towards reporting (*p*-value < 0.001) of child maltreatment. There is no significant difference in the scores of other subscales. The change in knowledge, attitude, and practice of medical students toward child maltreatment during the internship course is shown in Table 2.

**Table 2:** Change of knowledge, attitude, and practice of medical students toward child maltreatment during internship course

		**Start of internship course**		**End of internship course**		***P*-Value**
		**Mean**	**Standard Deviation**	**Mean**	**Standard Deviation**	
**Knowledge**	**Prevention**	12.65	2.48	13.38	2.05	0.001
	**Diagnosis**	18.97	3.5	19.1	2.94	0.45
	**Treatment**	1.55	0.57	1.59	0.56	0.41
	**Reporting**	3.89	1.95	3.74	0.76	0.89
**Attitude**	**Prevention**	5.96	0.93	5.84	0.82	0.1
	**Diagnosis**	10.22	1.47	9.83	1.34	0.001
	**Treatment**	1.73	0.28	1.7	0.26	0.26
	**Reporting**	4.5	1.93	3.84	1.56	< 0.001
**Practice**	**Prevention**	0.15	0.4	0.3	0.46	< 0.001
	**Diagnosis**	0.33	0.66	0.38	0.5	0.08
	**Treatment**	0.15	0.77	0.17	0.31	0.07
	**Reporting**	0.29	0.64	0.27	0.47	1
	**General**	0.68	1.06	1.12	1.08	< 0.001

### Comparison of knowledge, attitude, and practice of medical practitioners towards child maltreatment in terms of their sex and marital status

As there are differences in involvement and preoccupation with children by sex [46] and marital status [47], especially in Iranian culture [48], we examined the effect of sex and marital status on the knowledge, attitude, and practice of medical practitioners towards child maltreatment.

There is a significantly greater increase in the mean scores of female participants in comparison with male subjects in the subscales of the practice of diagnosis (*p*-value = 0.04) and treatment (*p*-value = 0.003) of child maltreatment during the internship course. Although, there is no significant difference between the scores of other subscales.

There is a significantly greater increase in married subjects’ mean scores compared with single participants in the subscales of the practice of treatment (*p*-value = 0.02) during the internship course. Although, there is no significant difference between the scores of other subscales.

## Discussion

Our study aimed to identify Iranian recently graduated medical physicians' knowledge, attitude, and practice toward child maltreatment and its change during the internship course.

The results of our study indicated a satisfactory level of knowledge and attitude toward child maltreatment among physicians. Our findings in the level of knowledge and attitude are higher than reported in physicians in Egypt (41%) [45] and community health workers in Tabriz, Iran [39]. This comparison shows better knowledge and attitude towards child maltreatment among physicians in the present time than four years ago in the same country and better than a country with a relatively similar culture to Iran.

The majority of the participants correctly recognized unwanted pregnancies and young age of parents, history of abuse in a parent, and the disability in children as child maltreatment risk factors. They agreed that child abuse can occur in high socioeconomic groups and is not primarily associated with poverty. These results are much higher than those of family physicians in France [49].

In diagnosing the signs and symptoms of child maltreatment, participants’ knowledge was sufficient in almost all questions. Although, 59.1% of participants detected the bruises over bony prominences as a sign of abuse and didn’t know that most such injuries are unintentional [50, 51]. Even so, this number is much lower than reported by physicians in Egypt [45].

These similar comparisons with a culturally similar country like Egypt and a country with different economic status and cultural atmosphere like France are a point of interest and can be the focus of further studies.

In the subscale of Attitude towards prevention of child abuse, nearly all of the participants strongly agreed/ agreed with the importance of child abuse education, which is in agreement with results reported among physicians in the USA [42], Sri Lanka [52], and higher than physicians in Gujarat, India [40].

By comparing our data in the subscales of knowledge and attitude with studies in other countries, it seems that Iranian physicians have equal or better knowledge and attitude regarding child maltreatment that can be promising and hopeful.

The practice skills of Iranian physicians regarding child maltreatment were the most conflictual area, and our findings show less experience than reported in physicians in Egypt [45] and Sri Lanka [52] and pediatricians in Kuwait [36]. The main reasons for underdiagnosis and underreporting are the lack of knowledge or the negative attitude [45]. This immense lack of experience among Iranian interns regarding child maltreatment, particularly despite the higher scores in attitude and knowledge, can cause a limitation in interpreting this.

The number of diagnosing and reporting a case of child abuse suggests that 18.6% of the participants diagnosed a case of maltreatment but did not report it. Although, 90.2% of the participants mentioned that they have no history of not reporting child abuse. It suggests that 8.8% of the participants were not completely sincere in answering the questionnaire. This matter and the lower rate of diagnosis and reporting (despite the satisfactory level of knowledge and attitude) suggest the stigma of child abuse among Iranian physicians. Reporting child maltreatment is not mandated in Iran, and there is no fixed strategy. Some of the cases are referred to the police, and some cases are reported to welfare organizations. In addition, social, cultural, and religious contexts cause many challenges in reporting abuse cases [19].

At the beginning of our study, only 7 (3%) of our participants formerly received any education about child maltreatment. This result is the same as the study of Li et al. [34] in China and the study of Pelletier and Knox in America [21]. There are some differences between our study and the studies which report higher former education: the subjects of some studies are not physicians, nor are the participants medical students [40, 45, 53]. In particular, the study of Sahebihagh et al. [39] performed in Tabriz, Iran and reported 26.99% of former education. The participants of this study are not physicians, with a mean age of 39 ± 7.85 and 14 ± 8.14 years of work experience.

We have evaluated the change of knowledge, attitude, and practice of medical students toward child maltreatment during the internship course (the last 18 months of medical training in Iran and medical students work as interns in teaching hospitals).

There was no significant difference in five of the eight knowledge and attitude subscales. The knowledge of the diagnosis of medical students improved significantly during the internship course. Although, the scores of attitudes toward diagnosis and reporting significantly decreased. In addition, the scores of the practice of prevention and general practice improved during the internship course.

Even though the level of knowledge and attitude among medical students are satisfactory after the internship period, our results have shown that the change in its level is insufficient. Especially the fact that the attitude of medical students decreased is a concerning point. One of the possible reasons can be the difference and paradox that medical students encounter between what they theoretically have learned and what is happening in teaching hospitals. Many ethical, legal, and cultural challenges regarding child maltreatment happen in the medical workplace [19]. Despite what medical students have learned in theory, they will face uncertainty about the child's future, reporting results, welfare centers' situation, and welfare organizations' capability [19].

On the other hand, despite significant improvement in two of five subscales of the practice of medical students, the score of the practice at the end of the internship course is not yet satisfactory. The main questions in the practice of prevention and general practice subscales are about the training courses regarding child maltreatment. The scores of the other subscales which mainly are about diagnosing, treatment, and reporting (which are aspects that are happening in the practice of a physician) are not changed during the internship course.

In addition to what has been mentioned, legal problems are other difficulties in practicing and even educating medical students regarding child maltreatment. According to Iran’s law, children’s guardians are their fathers, and fathers have the right to discharge the under 18 years old children against medical advice (unless there is an urgent, life-threatening matter) [54]. In many cases, the abusive father can discharge the child, and as reporting of child maltreatment is not mandatory in Iran, the child abuse reporter is not supported by the law [54]. On the other hand, in Iranian culture father or husband is the head of the family and the decision maker, and the children grow up with the value of being a good child for the parents [55].

All these obstacles and their paradox with what medical students learn theoretically before working in hospitals interfere with their education and practice.

There is a significant difference between the change of the scores of the subscale of the practice of diagnosis and treatment during the internship course in terms of the sex of participants (with better performance of female subjects). This result is congruent with the study of Sanyuz [56] which the majority of the physicians who made a child abuse diagnosis were females. The different roles of females regarding children might explain this difference. Due to their conventional role, females are more concerned and involved with children [57].

There is a significant difference between the change of the scores of the subscale of the practice of treatment during the internship course in terms of the marital status of participants (with better performance of married subjects). In the study of Kara et al. [41] among physicians in Ankara, it has been shown that the knowledge of married physicians regarding child maltreatment is significantly higher than that single physicians. Still, there is no examination of the level of practice of physicians regarding child abuse in terms of their marital status in other studies. This difference can have multiple reasons: the difference in involvement and preoccupation with children between married and single people, as childbearing is considered a value in Iranian culture [58], higher empathy in married people [59], and higher empathic skills (such as sharing and helpfulness, and internalizing the moral values) in married couples [60].

### Limitations and suggestions

With an adequate sample size and following the same subjects for 18 months, we tried to minimize the limitations. Although our participants were from one university in Tehran, the capital city of Iran, it may not represent the whole country. Iran University of Medical Sciences is a public university with students from all parts of the country which can decrease the limitation. However, this university is one of the top-ranked universities in Iran, and the results may differ in other institutes.

One of our limitations was the lack of experience regarding child maltreatment among Iranian interns. This immense lack of experience among Iranian interns regarding child maltreatment, particularly despite the higher scores in attitude and knowledge, can cause a limitation in interpreting our data, and the findings need to be considered very cautiously.

Other limitations in interpreting our data regarding the training and experience of participants were the relatively short duration of pediatric training in the internship period (three of 18 months), high (more than two-thirds) reports of no child maltreatment training session in the last three years despite scheduled training courses in students’ curriculum, and lack of related direct clinical experience.

Another limitation of our study is that we used a self-reported questionnaire to gather the data.

For further research, we suggest multicenter studies from different parts of the country, evaluating the role of experience by evaluating the variable of “years of practicing” in Iranian physicians, evaluating the efficacy of medical training regarding child maltreatment with a focus group discussion (FGD) or Delphi techniques or the Kirkpatrick model of training evaluation reaction, learning, behavior, and results, and investigating the exact flaws in medical training regarding child maltreatment.

## Conclusion

The results of our study reveal that the knowledge and attitude of Iranian physicians regarding child maltreatment are satisfactory. Although, the practice level has deficiencies and needs more education. In addition, our findings show that Iranian interns have a shallow experience regarding child maltreatment, particularly despite the higher scores in attitude and knowledge, which can be the reason for deficiencies in the practice level.

The stigma of child abuse among Iranian physicians, the lack of a uniform strategy for reporting child maltreatment in Iran, some social, cultural, and religious contexts, and the paradox between theoretical education and the common practice in educational hospitals are some of many obstacles and difficulties considering the education of Iranian interns regarding child maltreatment that further studies should focus on.

## Supplementary Information

**Additional file 1.**

**Additional file 2.**

## Data Availability

The datasets used and/or analyzed during the current study are available as a supplementary files.

## Abbreviations

WHO: World Health Organization
CI: Confidence interval
