# Supplementary material for: Knowledge, attitude, and practice regarding child maltreatment amongst Iranian medical students through internship course: an 18-month longitudinal study
Source: BMC Prim Care. 2023 Jan 31;24:37. doi: 10.1186/s12875-023-01988-9 (PMC9886537; doi:10.1186/s12875-023-01988-9)
Supplement: Supplementary file 1 — Additional file 1. [file 12875_2023_1988_MOESM1_ESM.docx]

Frequency Distribution of Participants’ Answers, in the End of Internship Course

| **Knowledge of medical practitioners about child maltreatment** | | | | | | | |
| --- | --- | --- | --- | --- | --- | --- | --- |
| **Knowledge of prevention questions** | | | | | | **Yes (%)** | **No (%)** |
| Prevention of unwanted pregnancies can reduce the risk of child abuse | | | | | | 95.7* | 4.3 |
| Child abuse prevalence is higher in forced marriages or very young couples | | | | | | 94.5* | 5.5 |
| Talking to the child about sexual abuse can cause fear or perversion | | | | | | 11.9 | 88.1* |
| To prevent child abuse, educating the children is more effective than educating the families | | | | | | 52.8 | 47.2* |
| The risk of exposure to abuse in a child with anomaly and disability is higher compared to other children | | | | | | 92.3* | 7.7 |
| Financial support of low-income families can prevent child abuse | | | | | | 91.1* | 8.9 |
| In families with broader social relationships, children are more likely to be abused | | | | | | 22.1 | 77.9* |
| Parents who have been abused as children themselves are less likely to abuse their children. | | | | | | 17.4 | 82.6* |
| **Knowledge of diagnosis questions** | | | | | | **Yes (%)** | **No (%)** |
| Children who have been abused, usually tell someone soon after the abuse | | | | | | 1.7 | 98.3* |
| Emotional and psychological signs of abuse may include fear of going home or of the parents | | | | | | 88.1* | 11.9 |
| A history that is vague and differs every time the parent tells it is a possible indicator of abuse | | | | | | 96.6* | 3.4 |
| The abuser in most cases is someone the child knows well | | | | | | 81.7* | 18.3 |
| Child abuse and neglect rarely occur amongst middle- or high-income earners | | | | | | 31.5 | 68.5* |
| Bruises over bony prominences are suspicious of abuse (e. g., chin, elbows, and knees) | | | | | | 59.1 | 40.9* |
| Burns are associated with many child-abuse cases | | | | | | 56.2* | 43.8 |
| If a child who has gained toilet education starts to wet his/her bed again, this may be a finding of child abuse | | | | | | 91.1* | 8.9 |
| **Knowledge of treatment questions** | | | | | | **Yes (%)** | **No (%)** |
| In treatment of child abuse | | Group and family therapies are most important | | | | 89.8* | 10.2 |
|  |  | Drugs have no use | | | | 30.2 | 69.8* |
| **Knowledge of reporting questions** | | | | | | **Yes (%)** | **No (%)** |
| Physicians are morally responsible to report any sign of child maltreatment | | | | | | 96.6* | 3.4 |
| Even with high suspicion of child maltreatment, physicians can not report it without the family consent | | | | | | 9.4 | 90.6* |
| **Attitude of medical practitioners towards child maltreatment** | | | | | | | |
| **Attitude towards prevention of child abuse statements** | | | **Strongly Agree %** | **Agree**  **%** | **Neutral**  **%** | **Disagree**  **%** | **Strongly Disagree %** |
| It is really important to educate physicians about child abuse | | | 71.1 | 26.4 | 2.6 | 0 | 0 |
| It is important to revise the laws about child abuse to prevent it | | | 64.7 | 29.4 | 4.7 | 1.3 | 0 |
| I’d like to attend workshops or symposiums to improve my knowledge regarding child abuse | | | 40.9 | 45.5 | 10.6 | 2.6 | 0.4 |
| Due to children's fear and embarrassment of reporting child abuse, even allowing the child to contact child abuse prevention centers is ineffective in reducing its rate. | | | 27.7 | 40 | 20 | 10.2 | 2.1 |
| **Attitude towards diagnosis of child abuse statements** | | | **Strongly Agree %** | **Agree**  **%** | **Neutral**  **%** | **Disagree**  **%** | **Strongly Disagree %** |
| The basics of how to diagnose possible child abuse should be taught to physicians. | | | 63.4 | 33.6 | 2.6 | 0.4 | 0 |
| A child suspected of child abuse should be evaluated by a physician at the first visit. | | | 64.7 | 30.2 | 4.7 | 0.4 | 0 |
| Most physicians do not have enough time in their office to evaluate patients suspected of child abuse. | | | 16.2 | 53.2 | 23 | 7.7 | 0 |
| I prefer to refer a case suspected of child abuse to a pediatrician. | | | 18.3 | 34.5 | 29.4 | 16.6 | 1.3 |
| I prefer to refer a case suspected of child abuse to a psychiatrist | | | 40.4 | 46 | 11.5 | 2.1 | 0 |
| **Attitude towards treatment of child abuse statements** | | | **Strongly Agree %** | **Agree**  **%** | **Neutral**  **%** | **Disagree**  **%** | **Strongly Disagree %** |
| Teaching the family about child abuse is one of treatment ways | | | 48.1 | 43 | 8.1 | 0.9 | 0 |
| Psychological and social support for families is one of the ways to treat child abuse. | | | 50.2 | 44.7 | 4.7 | 0.4 | 0 |
| **Attitude towards reporting of child abuse statements** | | | **Strongly Agree %** | **Agree**  **%** | **Neutral**  **%** | **Disagree**  **%** | **Strongly Disagree %** |
| My reasons for not reporting suspicions case  of child abuse is | Possible effect on the child | | 14 | 32.8 | 26.8 | 21.7 | 4.7 |
|  | Fear of anger from parents and family | | 9.4 | 31.9 | 30.6 | 21.3 | 6.8 |
|  | No legal obligation or authority to report | | 6.4 | 22.6 | 31.9 | 26.4 | 12.8 |
|  | Possible effect on my practice | | 9.4 | 27.7 | 30.6 | 20.4 | 11.9 |
| **Practice of prevention questions** | | | | **Zero**  **%** | **One or two %** | **Three to five %** | **More than five %** |
| How many child-abuse conferences have you attended in the last three years? | | | | 70.2 | 29.4 | 0.4 | 0 |
| **Practice of diagnosis questions** | | | | **Zero**  **%** | **One or two %** | **Three to five %** | **More than five %** |
| How many suspected cases of child abuse have you identified in the past year? | | | | 70.6 | 28.1 | 1.3 | 0 |
| How many suspected cases of child abuse have you identified during your career? | | | | 63.4 | 31.9 | 4.7 | 0 |
| **Practice of treatment questions** | | | | **Zero**  **%** | **One or two %** | **Three to five %** | **More than five %** |
| How many suspected child-abuse cases have you referred in the last year? | | | | 84.7 | 14.9 | 0.4 | 0 |
| How many suspected cases of child abuse have you referred during your career? | | | | 81.3 | 18.3 | 0.4 | 0 |
| **Practice of reporting questions** | | | | **Zero**  **%** | **One or two %** | **Three to five %** | **More than five %** |
| How many suspected cases of child abuse have you reported in the past year? | | | | 86.4 | 13.6 | 0 | 0 |
| How many suspected cases of child abuse have you reported during your career? | | | | 82.1 | 17.4 | 0.4 | 0 |
| How many suspected child-abuse cases have you not reported in the past year? | | | | 88.5 | 10.6 | 0.4 | 0.4 |
| How many suspected cases of child abuse have you not reported during your career? | | | | 90.2 | 9.4 | 0 | 0.4 |
| **General practice questions** | | | | | | **Yes**  **%** | **No**  **%** |
| Have you been trained in child abuse before graduation? | | | | | | 55.3 | 44.7 |
| Have the methods you have been taught to diagnose child abuse been sufficient? | | | | | | 14.9 | 85.1 |
| Have you ever tried to talk to parents about preventing child abuse? | | | | | | 27.7 | 72.3 |

*Correct answer
